# Speed and Distance Redistribution—Lower Limb Power Strategy in Single-Leg-Approach Jumps

**DOI:** 10.3390/life16010160

**Published:** 2026-01-18

**Authors:** Wei-Hsun Tai, Hsien-Te Peng, Jian-Zhi Lin, Hai-Bin Yu, Po-Ang Li

**Affiliations:** 1 School of Physical Education, Quanzhou Normal University, Quanzhou 362000, China; dlove520@hotmail.com; 2 Graduate Institute of Sport Coaching Science, Chinese Culture University, Taipei 11114, Taiwan; pxd@ulive.pccu.edu.tw; 3 Department of Physical Education, Chinese Culture University, Taipei 11114, Taiwan; 4 Department of Physical Education, National Taiwan University of Sport, Taichung 404401, Taiwan; 5 Department of Civic Education and Leadership, National Taiwan Normal University, Taipei 106308, Taiwan

**Keywords:** approach running jump, joint power generation, approach velocity, reactive strength index (RSI), lower-limb biomechanics

## Abstract

This study systematically investigated the influence of approach kinematics on the subsequent kinetics and power production strategies during the approach to running jumps with a single leg (ARJSL). Twenty-five physically active male university students performed ARJSL trials under two prescribed approach speeds (fast and slow) and three approach distances (3, 6, and 9 m) in a 2 × 3 within-subjects design. Three-dimensional motion capture synchronized with force platform data was used to quantify jump height (JH), vertical touchdown velocity (TDv), reactive strength index (RSI), peak joint power (hip, knee, and ankle), and joint stiffness. Significant approach speed × distance interactions were observed for JH (*p* = 0.006), TDv (*p* < 0.001), RSI (*p* = 0.014), ankle stiffness (*p* = 0.006), and peak power generation at all lower-limb joints (all *p* < 0.034). The results demonstrate that changes in approach strategy systematically alter the distribution of mechanical power among the hip, knee, and ankle joints, thereby influencing the effectiveness of horizontal-to-vertical momentum conversion during take-off. Notably, RSI and ankle stiffness were particularly sensitive to combined manipulations of speed and distance, highlighting their value as neuromechanical indicators of stretch–shortening cycle intensity and joint loading demands. In conclusion, ARJSL performance depends on finely tuned, speed- and distance-specific biomechanical adaptations within the lower extremity. These findings provide a constrained, joint-level mechanical characterization of how approach speed and distance interact to influence power redistribution and stiffness behavior during ARJSL, without implying optimal or performance-maximizing strategies.

## 1. Introduction

Vertical jumping is a cornerstone of athletic performance in sports that demands explosive lower-limb power and precise neuromuscular coordination; examples of vertical jumping include basketball, volleyball, and track and field [1]. Jump height depends not only on the magnitude of force produced by the lower extremities but also on the efficiency of intersegmental coordination that allows for the effective transfer of mechanical energy into vertical propulsion [2,3]. Coordination, defined as the dynamic regulation of multiple-joint degrees of freedom (DOF), plays a critical role in optimizing joint torque production and energy flow throughout the kinetic chain [4].

A commonly used movement pattern in these sports is an approach to running jumps with a single leg (ARJSL), in which an athlete employs a running approach before executing a one-leg take-off [5]. The running approach introduces horizontal momentum that must be efficiently converted into vertical lift during take-off, a mechanism fundamental to maximizing jump performance [6]. Prior studies have shown that increased approach speed can significantly influence ground reaction force (GRF), reduce ground contact time (CT), and increase joint torque output and muscle activation levels [7,8], thereby improving jump height and mechanical efficiency. Furthermore, this dynamic movement inherently utilizes the stretch–shortening cycle (SSC), which has been established to enhance muscle power, rate of force development (RFD), and neuromuscular control, through rapid eccentric–concentric coupling [9,10,11]. The effective utilization of the SSC in the single-leg jump relies heavily on the appropriate modulation of lower-limb stiffness [12,13].

Despite these advances, a critical gap remains in our comprehensive understanding of how approach distance and speed interact to influence ARJSL biomechanics. Most prior work has examined these parameters in isolation or focused primarily on landing mechanics and injury risks, rather than on optimizing performance across various approach strategies. For instance, while initial studies explored the influence of different approach parameters, finding that a 6 m approach distance might elicit optimal muscle activation under certain speed constraints [7], this preliminary work lacked the systematic and continuous manipulation necessary to identify the true optimal balance between horizontal momentum conversion and coordinated take-off mechanics [14]. Although temporal and force-related variables are known to account for a large proportion of variance in jump height [15], the modulation of these kinetic and kinematic factors under systematically varying approach conditions remains largely unexplored. Furthermore, recent biomechanical advances, such as those emphasizing the value of vertical jump metrics for assessing robust knee joint function [16], underscore the need to apply these precise analytical tools to examine the underlying mechanical adjustments (e.g., joint power contributions and segmental work) that occur under varied approach conditions.

Understanding how approach parameters influence single-leg jumping performance holds both theoretical and practical significance. Theoretically, it can elucidate the fundamental joint mechanisms underlying momentum transfer, stiffness regulation, and energy flux during dynamic unilateral take-off. Practically, the findings can directly inform the design of evidence-based training and competitive strategies by identifying the optimal approach conditions that maximize jump performance while minimizing the risk of mechanical inefficiency or overuse injuries.

Therefore, the purpose of this study was to systematically describe how variations in approach speed and distance are associated with changes in joint-level power generation and stiffness during ARJSL under controlled conditions. We hypothesized that approach mechanics modulate single-leg vertical jump outcomes through distinct joint-specific adaptations. Specifically, while higher speeds may increase power output and reduce contact time, we hypothesized that moderate approach distances provide the necessary temporal window to redirect horizontal momentum into vertical impulse. We further expected joint-dependent adaptations in stiffness and kinetics, where distal joints manage the increased demands of fast approaches during braking, and proximal joints contribute more significantly to propulsion during moderate conditions. This integrated analysis of joint and mechanical stiffness illuminates the mechanical strategies of momentum conversion, offering a more nuanced interpretation of inter-joint control in unilateral jumping.

## 2. Materials and Methods

### 2.1. Participants

Twenty-five male physical education students (age: 21.9 ± 1.5 years; height: 1.80 ± 0.06 m; mass: 71.9 ± 8.2 kg) voluntarily participated in this study. All participants were physically active and had engaged in regular sports training (≥3 sessions per week) involving jumping and sprinting tasks, such as basketball, volleyball, or track and field, for at least three years. None reported any lower-extremity musculoskeletal injury within the preceding six months. All participants were familiar with the single-leg-approach vertical jump (ARJSL) technique through formal coursework in sport biomechanics. The study was conducted in accordance with the Declaration of Helsinki and approved by the Institutional Review Board of National Taiwan University (protocol code: 2014ES086). All participants were informed of the experimental procedures and provided written consent prior to participation.

### 2.2. Experimental Design and Procedures

Before data collection, participants completed a standardized 10 min warm-up consisting of 5 min of treadmill running followed by 5 min of dynamic stretching of the lower-limb muscles. They were allowed sufficient practice trials to ensure familiarity with the experimental tasks. Participants wore standardized footwear (Mizuno K1GA140009, Lurng Furng, Inc., Taipei, Taiwan) throughout the tests to minimize variability due to footwear properties. Each participant performed ARJSLs under six approach conditions: two approach speeds (fast, >4.0 m/s; slow, <4.0 m/s) × three approach distances (3 m, 6 m, and 9 m). The order of conditions was randomized to mitigate fatigue or learning effects.

**Figure 1:**
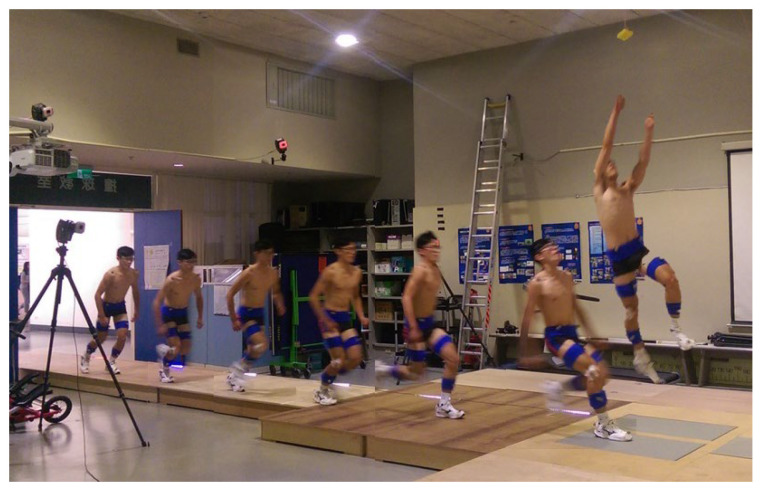
The ARJSL action diagram.

**Figure 2:**
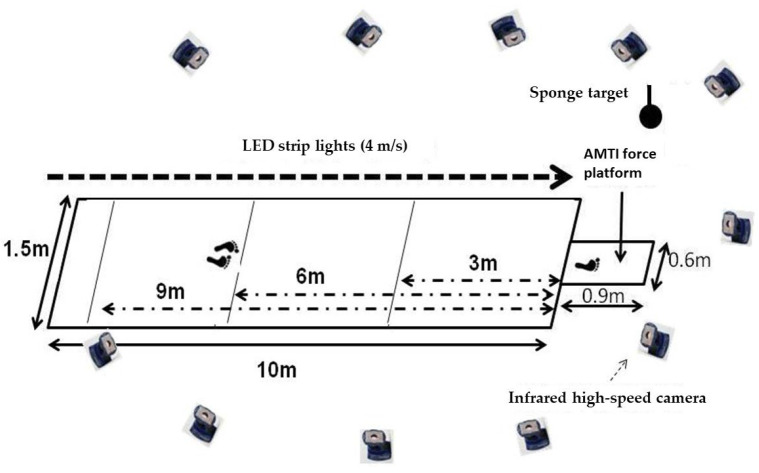
The experiment setting. Footprints illustrating the start position (at 6 m condition) and takeoff transition.

Approach speed was regulated using a visual pacing system consisting of an LED light bar positioned parallel to the runway, with the LED lights moving at a fixed speed of 4.0 m/s. Participants were instructed to run either faster or slower than the LED pace during the approach to achieve the desired submaximal or near-maximal conditions, rather than matching a precise target velocity. To verify the actual approach speed, the horizontal velocity of the sacrum marker at initial ground contact was calculated for each trial. The 4.0 m/s threshold for classifying approach speeds was adopted from the previous literature, which identified it as the approximate transition velocity between submaximal and near-maximal sprint phases during jump approaches [17,18,19]. Similarly, 6 m was defined as the moderate approach distance according to the reported range (3–9 m) typically used in approach jump tasks among trained male athletes [20,21]. Each condition included three valid trials, with a 1 min rest between trials and 5 min rest between conditions. A trial was considered valid only if the following conditions were fulfilled: (1) the participant contacted the force plate cleanly with the take-off leg, (2) there was no loss of balance or premature step adjustment, and (3) the participant performed a vertical jump without horizontal deviation exceeding 0.3 m. Invalid trials were repeated. The testing movement and experiment setting are shown in Figure 1 and Figure 2.

**Figure 3:**
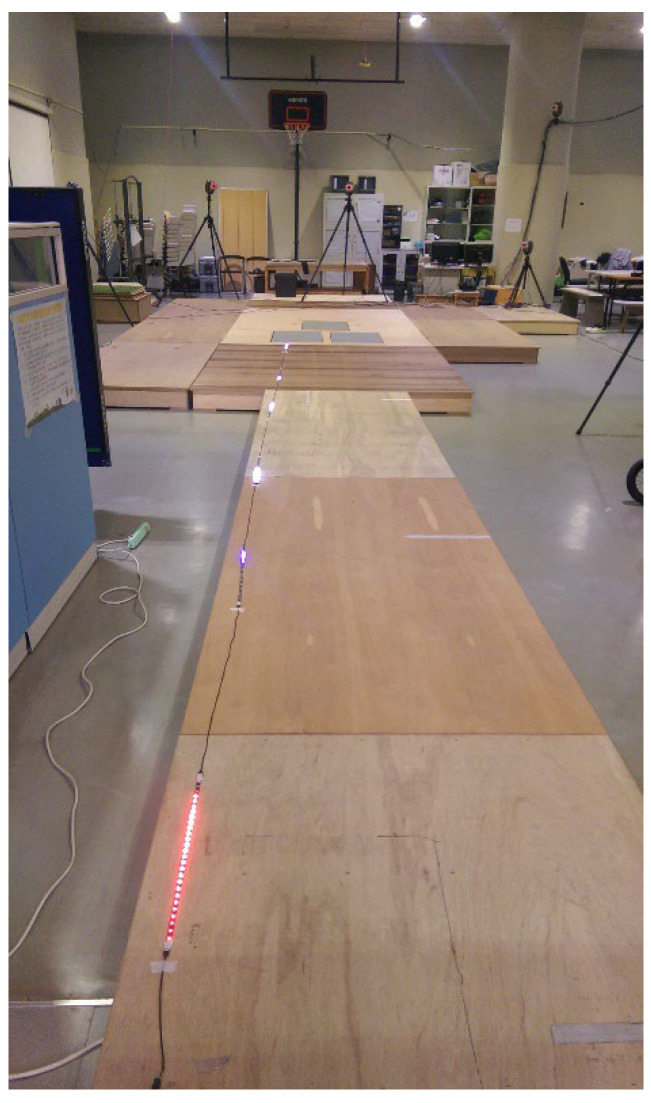
The experiment setting: 10 m wooden runway with 9 m of LED strip lights with a speed of 4 m/s beside it.

A 10 m wooden runway (1.5 m width) led to the force platform. An LED light bar system synchronized to 4 m/s was positioned beside the runway to aid in controlling approach speed (Figure 3). A soft ceiling sponge target was fixed at 125% of the participant’s maximal countermovement jump height (with arm reach) to motivate maximal effort and ensure consistent vertical jump execution.

### 2.3. Data Acquisition and Processing

Kinematic data were collected using an eleven-camera infrared motion capture system (Motion Analysis Corporation, Santa Rosa, CA, USA) at a sampling rate of 200 Hz, while ground reaction forces were recorded using a force platform (AMTI, Watertown, MA, USA) at 2000 Hz. Both systems were synchronized via EVaRT software (version 5.0.1, Motion Analysis Corp.). A 23-marker set based on the modified Helen Hayes model was used to define body segments and joint centers. Markers (19 mm diameter) were placed on the following anatomical landmarks: bilateral anterior and posterior superior iliac spines, C7, T10, sacrum, acromions, thighs, lateral and medial femoral epicondyles, shanks, lateral and medial malleoli, calcanei, and second metatarsal heads. The system was calibrated before each session, and static calibration trials were conducted to ensure that reconstruction errors were <1 mm. The dominant leg—defined as the preferred kicking leg [22]—was used for analysis. This selection was made to control for inter-limb asymmetrical effects and maintain analytical consistency.

Kinematic and kinetic data were processed using the MotionMonitor software (Motion Monitor V9, Innovative Sports Training, Chicago, IL, USA). Raw marker trajectories were low-pass filtered using a fourth-order zero-lag Butterworth filter with a 15 Hz cutoff frequency for kinematic data and 50 Hz cutoff frequency for GRF data. The cutoff frequencies were determined based on residual analysis to minimize signal distortion while retaining true biomechanical fluctuations [23]. Body segment parameters (mass, center of mass, and moment of inertia) were estimated using Dempster’s anthropometric tables. Joint moments were calculated via inverse dynamics and normalized to body weight.

### 2.4. Variable Definitions

The ground contact phase of the ARJSL was defined as the period between initial foot contact and toe-off from the force platform. A 20 N threshold of vertical ground reaction force (GRF) was used to determine the instants of contact and take-off. The horizontal velocity of the sacrum marker at initial ground contact was defined as the touchdown velocity (TDv). The impulse was obtained by integrating the vertical GRF–time curve during the ground contact phase, reflecting the total mechanical momentum applied to the body (see Equation (1)). The loading rate (LR) was computed as the average slope of the vertical GRF from initial contact to the peak GRF, indicating the rapidity of force development during impact absorption [24] (see Equation (2)). The reactive strength index (RSI) was calculated as flight time (FT)/ground contact time [25] (see Equation (3)). Flight time was defined as the time interval between toe-off and landing. Joint powers of the hip, knee, and ankle were calculated as the product of the joint moment and the corresponding joint angular velocity [26] (see Equation (4)). Positive power values represent energy generation by muscles, while negative values reflect energy absorption through eccentric contractions. The joint stiffness for each lower-limb joint was quantified as the ratio between the peak joint moment and the corresponding peak joint angular displacement during the eccentric phase, representing the resistance of the joint to angular deformation under load [27] (see Equation (5)). This measure reflects the overall mechanical behavior of the joint during eccentric loading rather than intrinsic material stiffness, as temporal factors, such as angular velocity and phase duration, are not explicitly captured. To examine inter-joint coordination, the relative contributions of hip, knee, and ankle joints to total positive mechanical power generation were calculated and expressed as a percentage of total power output. Ground reaction forces, impulses, and joint powers were normalized to each participant’s body weight to facilitate interindividual comparison.

(1) $$Impulse=\frac{1}{BW}\int_{t1}^{t2}F_{z}(t)dt$$

(2) $$LR=\frac{F_{peak}-F_{Ic}}{t_{peak}-t_{IC}}/BW$$

(3) $$RSI=\frac{t_{toeoff-landing}}{t_{ground\;contact}}$$

(4) P=M×ω

(5) $$K=\frac{M_{peak}}{\theta_{peak}}$$

### 2.5. Statistical Analysis

Data were analyzed using a two-way repeated-measures ANOVA to examine the main effects of approach speed (fast vs. slow), approach distance (3 m, 6 m, and 9 m), and their interaction effects. Mauchly’s test was used to verify sphericity assumptions; Greenhouse–Geisser corrections were applied when they were violated. Normality and homogeneity of variances were confirmed using the Kolmogorov–Smirnov and Levene’s tests, respectively. When significant main or interaction effects were observed, Bonferroni-adjusted post hoc tests were used for pairwise comparisons. The level of significance was set at α = 0.05. Effect sizes were reported using partial eta squared (η^2^), interpreted as small (0.01), moderate (0.06), or large (0.14) [28].

## 3. Results

**Figure 4:**
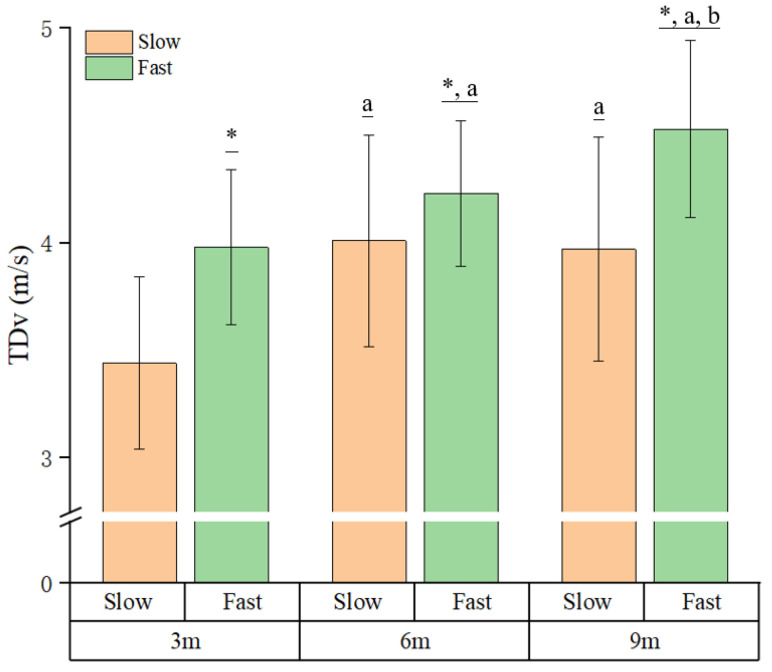
TDv (touchdown velocity) across approach speed (slow and fast) and distance conditions. * *p* < 0.05 (between the speeds); ^a,b^ *p* < 0.05 (between the distances: ^a^ = greater than 3 m and ^b^ = greater than 6 m).

**Figure 5:**
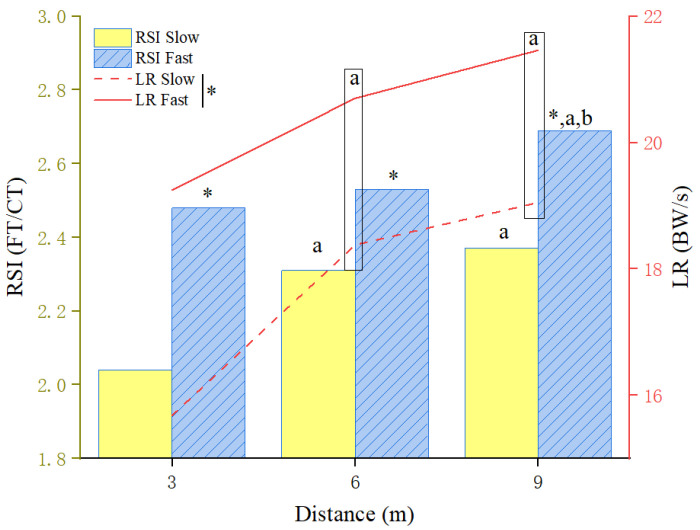
Effects of approach distance and speed on RSI and LR in ARJSL. * *p* < 0.05 (between the speeds); ^a,b^ *p* < 0.05 (between the distances: ^a^ = greater than 3 m and ^b^ = greater than 6 m); the narrow rectangles a = greater than 3 m (LR parameter); RSI = reactive strength index; LR = loading rate.

The two-way repeated-measures ANOVA revealed significant interaction effects between approach speed and distance on jump height, touchdown velocity, and reactive strength index (RSI) (*p* = 0.006, *p* < 0.001, and *p* = 0.014, respectively; Table 1 and Table 2 and Figure 4 and Figure 5). These findings indicate that the influence of approach speed on jumping performance varied depending on the approach distance.

### 3.1. Jump Height and Touchdown Velocity

Post hoc simple main effect analyses demonstrated that jump height was significantly greater during fast approach speeds compared with slow speeds at 3 m (*p* < 0.001, ES = 0.54) and 9 m (*p* = 0.025, ES = 0.19). Similarly, touchdown velocity increased significantly with faster approach speeds across all distances (3 m: *p* < 0.001, ES = 0.79; 6 m: *p* = 0.005, ES = 0.28; 9 m: *p* < 0.001, ES = 0.80). Moreover, distance also modulated performance: at slow speeds, jump height was greater at 6 m than 3 m (*p* = 0.003, ES = 0.22), and touchdown velocity increased progressively with longer approach distances at both slow and fast speeds (*p* < 0.001, ES = 0.57–0.59). To further evaluate the effectiveness and constraints of the LED-paced approach speed manipulation, the achieved touchdown velocities were examined across all speed and distance conditions (Figure 4). For the slow condition, the mean TDv increased from 3.44 ± 0.40 m/s at 3 m to 4.01 ± 0.49 m/s at 6 m, with comparable values observed at 9 m (3.97 ± 0.52 m/s). In the fast condition, the TDv progressively increased with approach distance, reaching 3.98 ± 0.36 m/s at 3 m, 4.23 ± 0.34 m/s at 6 m, and 4.53 ± 0.41 m/s at 9 m.

### 3.2. Reactive Strength Index and Loading Rate

The RSI exhibited a significant speed–distance interaction (Figure 5). Under slow approach conditions, RSI values at 9 m and 6 m were significantly higher than at 3 m (*p* < 0.001, ES = 0.49). At fast speeds, the RSI at 9 m exceeded that at both 6 m and 3 m (*p* = 0.005, ES = 0.20). Across all conditions, the RSI was consistently higher in fast approaches than in slow ones (3 m: *p* < 0.001; 6 m: *p* = 0.004; 9 m: *p* < 0.001). The main effect of speed was also significant for the loading rate, which was greater at fast approach speeds than at slow speeds (*p* < 0.001, ES = 0.58). Furthermore, the loading rate and vertical impulse both increased significantly with longer approach distances (9 m > 6 m > 3 m; *p* < 0.05).

### 3.3. Joint Power Generation

**Figure 6:**
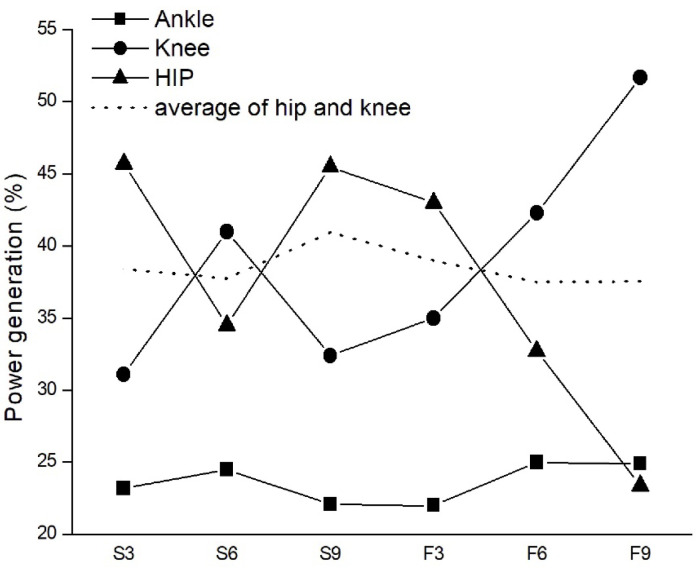
Effects of approach distance and speed on joint power generation in ARJSL. S = slow approach; F=fast approach; 3,6,9 = approach distance with m unit.

Significant interactions between approach speed and distance were also observed for hip, knee, and ankle power generation (Table 3 and Figure 6). Under slow speed conditions, hip power generation at 9 m exceeded that at 6 m (*p* = 0.023, ES = 0.15), whereas knee and ankle power generation were greatest at 6 m and 9 m, respectively (*p* < 0.05). In contrast, during fast approach speeds, hip power generation decreased at 9 m compared with slow approaches (*p* < 0.001, ES = 0.46), while knee and ankle power generation significantly increased at both 3 m and 9 m (knee: *p* < 0.001–0.001, ES = 0.36–0.48; ankle: *p* = 0.005, ES = 0.28). These findings suggest a shift in the power distribution strategy across joints with increasing approach speed, particularly highlighting greater reliance on distal joints under faster conditions.

**Table 3:** Results of joint power generation for each speed and distance.

	3 m	6 m	9 m	*η* ^2^	*p*
Hip power generation ^†^ (W/kg)					
Slow	17.6 ± 9.3	13.8 ± 11.1	20.6 ± 11.0 *^,b^	0.15	0.023
Fast	20.3 ± 12.9	13.2 ± 13.1	9.5 ± 7.4 *^,c^	0.27	0.001
Knee power generation ^†^ (W/kg)					
Slow	12.0 ± 5.4	16.4 ± 4.2 ^a^	14.7 ± 5.5	0.21	0.014
Fast	16.5 ± 5.4 *	17.1 ± 7.9	21.0 ± 8.4 *^,a,b^	0.23	0.007
Ankle power generation ^†^ (W/kg)					
Slow	8.9 ± 1.8	9.8 ± 2.0	10.0 ± 1.7 ^a^	0.16	0.034
Fast	10.4 ± 2.1 *	10.1 ± 2.0	10.1 ± 2.2	0.05	0.268

The significant difference was set as *p* < 0.05; ^†^ interaction found between the speeds and distances; * significant difference found between the speeds; ^a^, significantly greater than 3 m; ^b^, significance greater than 6 m; ^c^, significantly smaller than 3 m.

### 3.4. Joint Stiffness

**Figure 7:**
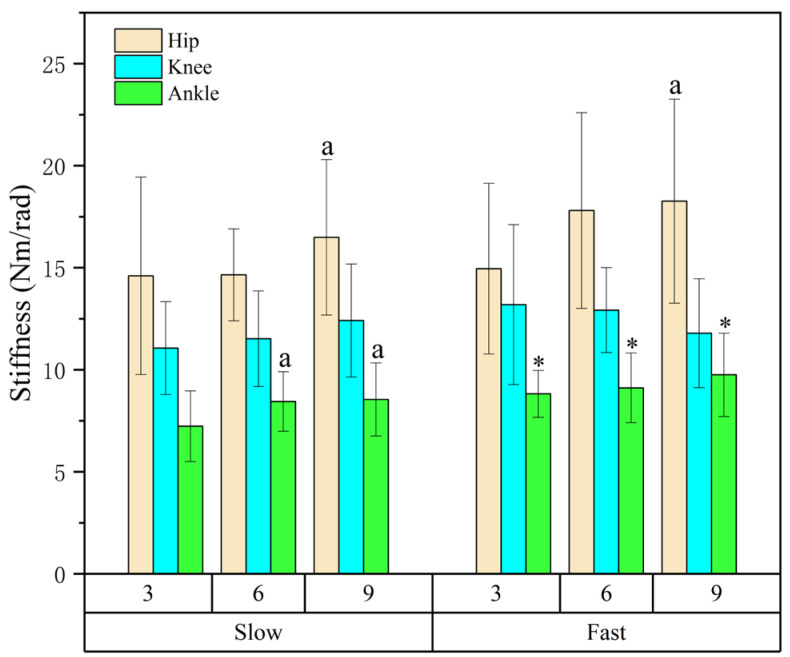
Effects of approach distance and speed on joint stiffness in ARJSL. * *p* < 0.05 (between the speeds)*;* ^a^ *p* < 0.05 (between the distances; ^a^ = greater than 3 m).

A significant interaction between speed and distance was found for ankle stiffness (*p* < 0.05), while hip stiffness was mainly affected by distance (Table 4 and Figure 7). Simple main effect tests revealed that ankle stiffness increased with faster approaches at all distances (3 m: *p* = 0.004, ES = 0.23; 6 m: *p* = 0.027, ES = 0.14; 9 m: *p* = 0.011, ES = 0.18). In addition, both hip and ankle stiffness values at 9 m were significantly greater than those at 3 m (*p* = 0.012, ES = 0.32; *p* = 0.006, ES = 0.19), indicating that a longer approach enhanced joint stiffness and load tolerance during take-off.

**Table 4:** Results of joint stiffness for each speed and distance.

	3 m	6 m	9 m	*η* ^2^	*p*
Hip stiffness (Nm/rad)					
Slow	14.60 ± 4.84	14.65 ± 2.25	16.49 ± 3.81 ^a^	0.32	0.012
Fast	14.95 ± 4.18	17.80 ± 4.80	18.26 ± 5.00 ^a^		
Knee stiffness (Nm/rad)					
Slow	11.06 ± 2.28	11.52 ± 2.34	12.41 ± 2.77	0.02	0.833
Fast	13.19 ± 3.92	12.92 ± 2.08	11.79 ± 2.67		
Ankle stiffness ^†^ (Nm/rad)					
Slow	7.23 ± 1.73	8.44 ± 1.46 ^a^	8.54 ± 1.79 ^a^	0.19	0.006
Fast	8.82 ± 1.15 *	9.11 ± 1.71 *	9.75 ± 2.04 *	0.05	0.192

The significant difference was set as *p* < 0.05; ^†^ interaction found between the speeds and distances; * significant difference found between the speeds; ^a^, significantly greater than 3 m.

## 4. Discussion

The present study examined how externally constrained approach speed and distance influence joint-level mechanical behavior during ARJSLs. Collectively, the results demonstrate that ARJSL performance is sensitive to approach mechanics, with approach speed being strongly associated with joint-specific variations in power and stiffness. The primary finding is that approach speed acted as the dominant factor shaping joint power distribution and quasi-stiffness patterns under controlled laboratory conditions. Importantly, these results describe task-dependent mechanical responses rather than optimized movement strategies or neuromuscular adaptations.

The achieved touchdown velocities demonstrated a clear group-level separation between the fast and slow conditions across all approach distances. Although partial overlap in TDv distributions was observed between adjacent conditions, particularly between slow–long and fast–short approaches, the prescribed speed categories remained distinguishable at the group level [29]. This may reflect individual differences in speed regulation under externally paced constraints rather than convergence toward a fixed or strictly controlled velocity. Collectively, these findings confirm that the LED pacing paradigm effectively biased approach speed at the group level while preserving natural variability in execution. Accordingly, the observed biomechanical responses should be interpreted as mechanical adaptations to externally biased approach conditions, rather than as precise outcomes of tightly constrained or fully self-selected running speeds.

Across the conditions, faster approaches were associated with higher touchdown velocity and an increased RSI. In the present study, the RSI reflects the temporal relationship between airborne and contact phases rather than being a direct indicator of neuromuscular performance [25]. The observed increase in the RSI under faster approach conditions was primarily driven by a reduction in contact time, while flight time exhibited comparatively smaller changes. This pattern indicates a mechanical timing adjustment in response to externally paced constraints that limit the duration available for force application during take-off.

Joint power analysis revealed a systematic redistribution of mechanical contribution across the lower limb with increasing approach speed. Specifically, hip power generation decreased while knee power increased, whereas ankle power remained relatively stable. This inverse pattern is interpreted as a task-dependent redistribution of joint-level mechanical workload at the group level, rather than evidence of a superior, optimized, or neuromuscularly driven coordination strategy [30]. Under externally paced and time-constrained conditions, reduced hip power may coincide with greater reliance on knee contribution to satisfy the mechanical demands of take-off, without implying diminished hip involvement or enhanced mechanical efficiency [31].

Ankle quasi-stiffness increased with faster approach speeds, indicating a greater resistance to angular displacement under elevated external loading conditions. This finding is consistent with the observed increases in touchdown velocity and reduced ground contact time, suggesting that the ankle joint operated under progressively higher mechanical demands as approach speed increased. Importantly, joint stiffness in the present study was quantified using a peak joint moment–angular displacement ratio during the eccentric phase and therefore represents a quasi-stiffness descriptor rather than true mechanical, elastic, or neuromuscular stiffness [27]. Within this framework, the observed increase in ankle quasi-stiffness reflects a systematic alteration in joint-level mechanical behavior in response to externally imposed task constraints, rather than an intrinsic modification of elastic properties or neural control strategies. From a performance perspective, higher ankle quasi-stiffness coincided with elevated RSI values and shorter contact durations, indicating that the ankle joint contributed to maintaining structural integrity and force transmission under time-constrained loading [32]. However, this behavior should be interpreted as a task-dependent mechanical response that supports rapid force application, rather than evidence of enhanced efficiency or optimized energy storage [30]. The absence of temporal stiffness metrics and electromyographic data precludes further mechanistic interpretation beyond joint-level mechanical behavior.

Consequently, the present results characterize lower-limb mechanical behavior under controlled, externally paced conditions rather than self-selected or competition-like jumping strategies. Accordingly, the ecological relevance and generalizability of these findings should be interpreted within the constraints of the experimental paradigm.

### 4.1. Practical Implications

The present results do not prescribe optimal approach strategies or training interventions. Rather, they provide descriptive benchmarks illustrating how joint-level mechanical variables, including RSI and ankle quasi-stiffness, respond to the systematic manipulation of approach speed and distance under controlled conditions in physically active individuals. Within this context, the RSI and quasi-stiffness may serve as evaluative descriptors of task-dependent mechanical responses to externally imposed approach constraints. These measures may support qualitative interpretation in future experimental or applied studies when manipulating approach-related task parameters, but they should not be interpreted as direct indicators of performance optimization or injury risk reduction based on the current data.

### 4.2. Limitations

Several limitations should be noted. The participants were physically active physical education students rather than elite athletes, and the results therefore reflect group-level mechanical trends rather than uniform individual adaptations. The absence of electromyographic data restricts interpretation to joint-level mechanical behavior. In addition, the externally paced LED approach constrained natural speed regulation, and the findings should be interpreted as reflecting lower-limb mechanics under controlled laboratory conditions rather than self-selected or competition-like jumping strategies.

## 5. Conclusions

This study examined how externally constrained approach speed and distance influence joint-level mechanical behavior during single-leg-approach run jumps under controlled laboratory conditions. The results indicate that approach speed is the primary factor associated with systematic changes in joint power distribution and ankle quasi-stiffness, whereas approach distance further modulates these mechanical responses by altering momentum-related demands. Faster approaches were characterized by reduced hip power contribution and increased knee involvement, accompanied by greater ankle quasi-stiffness, reflecting a task-dependent redistribution of mechanical workload rather than an optimized or superior movement strategy. Importantly, these findings represent group-level mechanical patterns observed under externally paced conditions and should not be interpreted as direct indicators of neuromuscular coordination, elastic energy utilization, or performance optimization. Collectively, the present results provide a descriptive mechanical framework for understanding how lower-limb joint behavior responds to controlled variations in approach conditions and offer a reference point for future investigations employing more ecologically valid task designs and integrated neuromuscular measurements.

## Figures and Tables

**Table 1:** Results of jump height and touchdown velocity for each speed and distance.

	3 m	6 m	9 m	*η* ^2^	*p*
JH ^†^ (cm)					
Slow	71 ± 11	75 ± 9 ^a^	74 ± 11	0.22	0.003
Fast	78 ± 8 *	76 ± 7	77 ± 10 *	0.16	0.140
TDv ^†^ (m/s)					
Slow	3.44 ± 0.40	4.01 ± 0.49 ^a^	3.97 ± 0.52 ^a^	0.57	0.000
Fast	3.98 ± 0.36 *	4.23 ± 0.34 *^,a^	4.53 ± 0.41 *^,a,b^	0.59	0.000

The significant difference was set as *p* < 0.05; ^†^ interaction found between the speeds and distances; * significant difference found between the speeds; ^a^, significantly greater than 3 m; ^b^, significantly greater than 6 m; JH = jump height; TDv = touchdown velocity.

**Table 2:** Results of RSI, LR, and impulse for each speed and distance.

	3 m	6 m	9 m	*η* ^2^	*p*
RSI ^†^ (FT/CT)					
Slow	2.04 ± 0.4	2.31 ± 0.35 ^a^	2.37 ± 0.46 ^a^	0.49	0.000
Fast	2.48 ± 0.46 *	2.53 ± 0.55 *	2.69 ± 0.61 *^,a,b^	0.20	0.005
LR (BW/s)					
Slow	15.68 ± 6.29	18.38 ± 6.10 ^a^	19.05 ± 6.53 ^a^	0.43	0.000
Fast	19.25 ± 6.06 *	20.70 ± 5.88 *^,a^	21.46 ± 6.49 *^,a^		
Vertical Impulse (BW·s)					
Slow	0.36 ± 0.05	0.36 ± 0.05	0.37 ± 0.05 ^a,b^	0.32	0.012
Fast	0.37 ±0.05	0.37 ± 0.05	0.38 ± 0.05 ^a,b^		
Horizontal Impulse (BW·s)					
Slow	0.20 ± 0.05	0.21 ± 0.04	0.21 ± 0.04	0.16	0.157
Fast	0.21 ± 0.04	0.22 ± 0.03	0.21 ± 0.04		

The significant difference was set as *p* < 0.05; ^†^ interaction found between the speeds and distances; * significant difference found between the speeds; ^a^, significantly greater than 3 m; ^b^, significantly greater than 6 m; BW = body weight, RSI = reactive strength index, FT = flight time, CT = contact time, and LR = loading rate.

## Data Availability

The original contributions presented in this study are included in the article. Further inquiries can be directed to the corresponding authors.

## References

[1] Lopez-Betancourt S., García-Torres C., López-Galvis D.A., Monsalve J.M., Rojas-Jaramillo A. (2025). Individualizing Strength Training Using the Vertical Force-Velocity Profile in Vertical Jumping and its Effect on Athletic Performance: A Systematic Review. Phys. Educ. Theory Methodol..

[2] He J., Li M., Zhang Q., Zhang Z. (2025). Associations between the performance of vertical jump and accelerative sprint in elite sprinters. Front. Bioeng. Biotechnol..

[3] Geantă V.A., de Hillerin P.J., Iacobini A.R., Camenidis C.M., Ionescu A. (2025). Differences in Average Power Output Values from Computational Models of Repeated Vertical Jump Tests: A Single-Group Quasi Experimental Approach. J. Funct. Morphol. Kinesiol..

[4] Dubois O., Roby-Brami A., Parry R., Khoramshahi M., Jarrassé N. (2023). A guide to inter-joint coordination characterization for discrete movements: A comparative study. J. Neuroeng. Rehabil..

[5] Tai W.H., Wang L.I., Peng H.T. (2018). Biomechanical Comparisons of One-Legged and Two-Legged Running Vertical Jumps. J. Hum. Kinet..

[6] Ricupito R., Bravi M., Santacaterina F., Campardo G., Guarise R., Castellucci R., Alaoui I.B., Forelli F. (2025). Biomechanical Alterations in the Unweight Phase of the Single-Leg Countermovement Jump After ACL Reconstruction. J. Funct. Morphol. Kinesiol..

[7] Tai W., Peng H., Lin J., Lo S., Yu H., Huang J. (2019). Biomechanical characteristics of single leg jump in collegiate basketball players based on approach technique. Appl. Sci..

[8] Chen C.-F., Wu H.-J. (2022). The effect of an 8-week rope skipping intervention on standing long jump performance. Int. J. Environ. Res. Public Health.

[9] Markovic G., Mikulic P. (2010). Neuro-musculoskeletal and performance adaptations to lower-extremity plyometric training. Sports Med..

[10] Golubić A., Šarabon N., Marković G. (2021). Association between trunk muscle strength and static balance in older women. J. Women Aging.

[11] Tai W.-H., Peng H.-T., Lin J.-Z., Li P.-A. (2025). Adaptive Neuromuscular Co-Contraction Strategies Under Varying Approach Speeds and Distances During Single-Leg Jumping: An Exploratory Study. Life.

[12] Brazier J., Maloney S., Bishop C., Read P.J., Turner A.N. (2019). Lower extremity stiffness: Considerations for testing, performance enhancement, and injury risk. J. Strength Cond. Res..

[13] Yu L., Mei Q., Xiang L., Liu W., Mohamad N.I., István B., Fernandez J., Gu Y. (2021). Principal component analysis of the running ground reaction forces with different speeds. Front. Bioeng. Biotechnol..

[14] Fahey J.T., Comfort P., Jones P., Ripley N.J. (2025). Effect of 6-week single leg countermovement jump training on force time metrics in elite female youth footballers. J. Sports Sci..

[15] Laffaye G., Wagner P.P., Tombleson T.I. (2014). Countermovement jump height: Gender and sport-specific differences in the force-time variables. J. Strength Cond. Res..

[16] Kotsifaki A., Van Rossom S., Whiteley R., Korakakis V., Bahr R., Sideris V., Jonkers I. (2022). Single leg vertical jump performance identifies knee function deficits at return to sport after ACL reconstruction in male athletes. Br. J. Sports Med..

[17] Takai Y., Miyazaki T., Sugisaki N., Yoshimoto T., Mitsukawa N., Kobayashi K., Tsuchie H., Kanehisa H. (2025). Spatiotemporal and kinetic characteristics during maximal sprint running in fast running soccer players. PLoS ONE.

[18] Loturco I., Contreras B., Kobal R., Fernandes V., Moura N., Siqueira F., Winckler C., Suchomel T., Pereira L.A. (2018). Vertically and horizontally directed muscle power exercises: Relationships with top-level sprint performance. PLoS ONE.

[19] Bridgett L. The effect of run-up speed on long jump performance. Proceedings of the 20 International Symposium on Biomechanics in Sports.

[20] Tai W.-H., Wang L.-I., Peng H.-T. (2018). Biomechanical comparisons of one-legged and two-legged running vertical jumps. J. Hum. Kinet..

[21] Asaeda M., Hirata K., Ohnishi T., Ito H., Miyahara S., Mikami Y. (2024). Differences in lower-limb biomechanics during single-leg landing considering two peripheral fatigue tasks. PLoS ONE.

[22] Beling J., Wolfe G.A., Allen K.A., Boyle J.M. (1998). Lower extremity preference during gross and fine motor skills performed in sitting and standing postures. J. Orthop. Sports Phys. Ther..

[23] Winter D.A. (2009). Biomechanics and Motor Control of Human Movement.

[24] Ueda T., Hobara H., Kobayashi Y., Heldoorn T., Mochimaru M., Mizoguchi H. (2016). Comparison of 3 methods for computing loading rate during running. Int. J. Sports Med..

[25] Northeast J., Russell M., Shearer D., Cook C.J., Kilduff L.P. (2019). Predictors of linear and multidirectional acceleration in elite soccer players. J. Strength Cond. Res..

[26] Ohji S., Aizawa J., Hirohata K., Ohmi T., Kawasaki T., Koga H., Yagishita K. (2024). Relationship between single-leg vertical jump and drop jump performance, and return to sports after primary anterior cruciate ligament reconstruction using hamstring graft. Int. J. Sports Phys. Ther..

[27] Hobara H., Kimura K., Omuro K., Gomi K., Muraoka T., Sakamoto M., Kanosue K. (2010). Differences in lower extremity stiffness between endurance-trained athletes and untrained subjects. J. Sci. Med. Sport..

[28] Cohen J. (2013). Statistical Power Analysis for the Behavioral Sciences.

[29] Padulo J., Ayalon M., Barbieri F.A., Di Capua R., Doria C., Ardigò L.P., Dello Iacono A. (2023). Effects of gradient and speed on uphill running gait variability. Sports Health.

[30] Shamsoddini A., Hollisaz M.T. (2022). Biomechanics of running: A special reference to the comparisons of wearing boots and running shoes. PLoS ONE.

[31] Lenhart R., Thelen D., Heiderscheit B. (2014). Hip muscle loads during running at various step rates. J. Orthop. Sports Phys. Ther..

[32] Wager J.C., Challis J.H. (2024). Mechanics of the foot and ankle joints during running using a multi-segment foot model compared with a single-segment model. PLoS ONE.

